# Genotype status and arrhythmic risk in sport participants with hypertrophic cardiomyopathy

**DOI:** 10.1093/ehjopen/oeag127

**Published:** 2026-08-18

**Authors:** Frédéric Myon, Mathilde Delatour, Nathalie Behar, François Carré, Erwan Donal, Frédéric Schnell

**Affiliations:** Department of Sports Medicine, University Hospital Pontchaillou, 2 rue Henri Le Guilloux, 35033 Rennes, France; Department of Cardiology, University Hospital Pontchaillou, 2 rue Henri Le Guilloux, 35033 Rennes, France; Department of Sports Medicine, University Hospital Pontchaillou, 2 rue Henri Le Guilloux, 35033 Rennes, France; Department of Cardiology, University Hospital Pontchaillou, 2 rue Henri Le Guilloux, 35033 Rennes, France; Department of Cardiology, University Hospital Pontchaillou, 2 rue Henri Le Guilloux, 35033 Rennes, France; CIC 1414, INSERM, University Hospital Pontchaillou, University of Rennes, 2 rue Henri Le Guilloux, 35033 Rennes, France; Department of Sports Medicine, University Hospital Pontchaillou, 2 rue Henri Le Guilloux, 35033 Rennes, France; CIC 1414, INSERM, University Hospital Pontchaillou, University of Rennes, 2 rue Henri Le Guilloux, 35033 Rennes, France; LTSI, INSERM, U1099, University of Rennes, 263 Avenue du Général Leclerc, 35042 Rennes, France; Department of Cardiology, University Hospital Pontchaillou, 2 rue Henri Le Guilloux, 35033 Rennes, France; CIC 1414, INSERM, University Hospital Pontchaillou, University of Rennes, 2 rue Henri Le Guilloux, 35033 Rennes, France; LTSI, INSERM, U1099, University of Rennes, 263 Avenue du Général Leclerc, 35042 Rennes, France; Department of Sports Medicine, University Hospital Pontchaillou, 2 rue Henri Le Guilloux, 35033 Rennes, France; CIC 1414, INSERM, University Hospital Pontchaillou, University of Rennes, 2 rue Henri Le Guilloux, 35033 Rennes, France; LTSI, INSERM, U1099, University of Rennes, 263 Avenue du Général Leclerc, 35042 Rennes, France

**Keywords:** Hypertrophic cardiomyopathy, Athletes, Exercise, Genetic, Ventricular arrhythmia

## Abstract

**Aims:**

The impact of genotype on arrhythmic risk in sports-active hypertrophic cardiomyopathy (HCM) patients remains unclear. We compared outcomes between genotype-positive (G+) and genotype-negative (G−) athletes with HCM.

**Methods and results:**

In this retrospective study, HCM patients actively engaged in sports underwent standardized baseline evaluation and follow-up. The primary endpoint included sudden cardiac death (SCD)/sudden cardiac arrest, appropriate implantable cardioverter-defibrillator (ICD) therapy, sustained ventricular tachycardia/non-sustained ventricular tachycardia (NSVT). Secondary outcomes included a composite of ventricular arrhythmias, atrial fibrillation, ischaemic stroke, and heart-failure hospitalization. Fifty-nine patients [age 40 years (23.2–50.0)] were followed for 6.3 years (3.0–12.6). Twenty-nine participants (49.2%) carried a likely pathogenic or pathogenic variant, most commonly MYBPC3 (44.8%), MYH7 (24.1%), and TNNT2 (17.2%). At inclusion, median sport practice was 6.0 (3.0–10.0) hours per week, with 67.8% of competitors. At last follow-up, 88.1% remained active in sports. The ESC 5-year SCD risk score was slightly higher in G+ compared with G− patients [1.87% (1.47–3.67) vs. 1.54% (1.24–2.20), *P* = 0.048], but most of the participants were classified as low risk. Ventricular arrhythmias occurred in 20.7% of G+ and 23.3% of G− patients (*P* = 0.807), with similar event-free survival (*P* = 0.685). Most ventricular arrhythmias were isolated NSVT (84.6%). Hard ventricular arrhythmic events were limited to two ICD-terminated ventricular fibrillations in G+ patients, one at rest and one during low-intensity exercise. The incidence of the composite secondary cardiovascular outcome was comparable between groups (31.0% vs. 33.3%, *P* = 0.850).

**Conclusion:**

In this exploratory cohort of sports-active HCM patients, ventricular arrhythmic and cardiovascular event rates were similar between G+ and G− participants during follow-up.

## Graphical Abstract

**Graphical Abstract:**
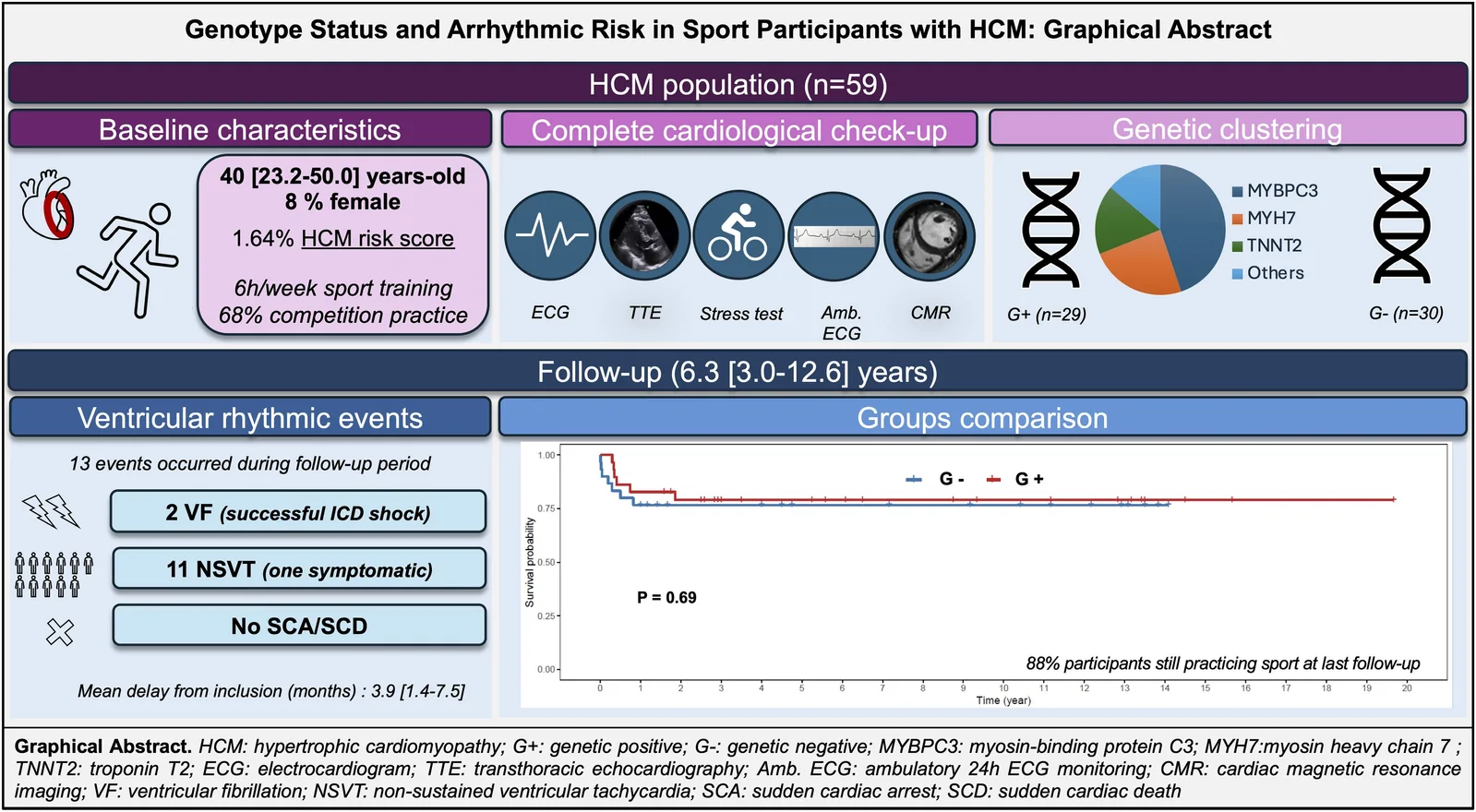

## Introduction

Hypertrophic cardiomyopathy (HCM) is the most common inherited cardiomyopathy and remains an important cause of exercise-related sudden cardiac arrest in young individuals.^1^ Contemporary studies nevertheless suggest that appropriately selected patients with HCM may participate in regular exercise and competitive sports with relatively low rates of adverse cardiovascular events.^2^ Pathogenic sarcomeric variants are identified in a substantial proportion of patients with HCM and may influence disease expression and clinical outcomes.^3^ However, whether genotype status provides additional prognostic information in physically active individuals with HCM remains uncertain.

We therefore aimed to compare arrhythmic and cardiovascular outcomes between genotype-positive (G+) and genotype-negative (G−) sports-active individuals with HCM.

## Methods

### Study population

We retrospectively included consecutive individuals ≥12 years old with HCM,^4^ who participate in regular sports activity (≥2 h/week) and were evaluated in a tertiary sports cardiology and cardiomyopathy centre. The study complied with the Declaration of Helsinki, received local ethics approval (*n*° 25.165). All subjects provided informed consent.

Baseline evaluation included clinical history, sports participation characteristics, electrocardiogram (ECG), echocardiography at rest and exercise, cardiopulmonary exercise test, ambulatory ECG monitoring, cardiac magnetic resonance imaging, and genetic testing. Patients carrying likely pathogenic or pathogenic (LP/P) genetic variants, i.e. Class 4 or 5 variants, were classified as G+. Participants underwent at least annual follow-up in our centre. The primary endpoint was a composite of ventricular arrhythmic events including sudden cardiac arrest/death (SCA/SCD), appropriate implantable cardiovert-defibrillator (ICD) therapy, sustained ventricular tachycardia (SVT), and non-sustained ventricular tachycardia (NSVT; ≥3 consecutive ventricular beats at >120 bpm lasting <30 s). NSVT was counted irrespective of symptoms. This endpoint was intended to capture ventricular arrhythmic expression in this exploratory sports-active HCM cohort, rather than hard arrhythmic events only. An analysis excluding NSVT was also performed to report hard ventricular arrhythmic events separately. Secondary outcomes included a broader composite of cardiovascular events comprising all components of the primary endpoint together with atrial fibrillation (AF), ischaemic stroke, and hospitalization for heart failure.

### Statistical analysis

Continuous variables were expressed as mean ± SD or median (interquartile range), and categorical as counts and percentages. Group comparisons used Student’s t-test, Mann–Whitney U test, χ^2^ test, or Fisher’s exact test, as appropriate. Kaplan–Meier analysis with log-rank testing was used for event-free survival. A two-sided *P* < 0.05 was considered statistically significant. Analyses were conducted using RStudio.

## Results

### Population characteristics

Fifty-nine participants were included [age 40.0 years (23.2–50.0), 13.6% were women; *Table 1*]. A LP/P genetic variant was identified in 29 patients (49.2%), most commonly involving MYBPC3 (44.8%), MYH7 (24.1%), and TNNT2 (17.2%). At baseline, symptoms were present in 33.9% of cases, with dyspnoea and palpitations being the most frequent, each reported in 13.6% of participants; ECG abnormalities were found in 81.4% of patients; maximal wall thickness was 15.0 mm (15.0–18.0), left ventricular outflow tract obstruction was present in 6.8%; late gadolinium enhancement in 44.1% [5.9% (2.9–8.8) of LV global mass when present]; NSVT in 7.4%. The ESC risk score for SCD at 5 years was 1.64% (1.34, 2.32), 88.1% of participants were classified as low-risk. Beta-blockers were prescribed in 25.4% of cases, three (5.1%) had an ICD for primary prevention, while two eligible patients declined implantation (one G+, one G−). At baseline, the mean weekly training volume was 6.0 h (3.0–10.0). Endurance (49.2%) and mixed (45.8%) sports were the dominant disciplines. Competitive sports participation was reported in 67.8% of participants. Peak VO2 was 38.5 ± 11.3 mL/min/kg.

**Table 1:** Baseline clinical characteristics of the population

	All participants (*n* = 59)	G+ group (*n* = 29)	G− group (*n* = 30)	*P*-value
Age (years)	40 (23.2–50.0)	27.5 (17.5–47.8)	42.4 (32.6–56.9)	0.019
Female, *n* (%)	8 (13.6)	4 (13.8)	4 (13.3)	1.00
**Sport participation**				
Hours of training/week	6.0 (3.0–10.0)	4.0 (3.0–10.0)	6.25 (3.0–10.0)	0.431
Competitive practice, *n* (%)	40 (67.8)	20 (69.0)	20 (66.7)	1.00
**Clinical characteristics**				
1^st^ degree FH of HCM, *n* (%)	18 (30.5)	13 (44.8)	5 (16.7)	0.019
1^st^ degree FH of SCD, *n* (%)	8 (13.6)	5 (17.2)	3 (10.0)	0.472
CV symptoms, *n* (%)	20 (33.9)	10 (34.5)	10 (33.3)	1.00
Abnormal ECG, *n* (%)	48 (81.4)	24 (82.8)	24 (80.0)	1.00
Beta-blocker, *n* (%)	15 (25.4)	8 (27.6)	7 (23.3)	0.708
ICD, *n* (%)	3 (5.1)	2 (6.9)	1 (3.3)	0.612
**Cardiac evaluation**				
Max LV WT (mm)	15.0 (15.0–18.0)	16.0 (15.0–19.0)	15.0 (15.0–17.0)	0.655
LVEDD (mm)	46.4 ± 5.7	44.2 ± 5.4	48.5 ± 5.3	0.003
LVEF (%)	68.2 ± 6.1	68.6 ± 6.0	67.8 ± 6.2	0.637
Apical HCM pattern, *n* (%)	9 (15.3)	1 (3.4)	8 (26.7)	0.026
LV obstruction (rest/exercise), *n* (%)	4 (6.8)	2 (6.9)	2 (6.7)	1.00
LGE present, *n* (%)	26 (44.1)	16 (55.2)	10 (33.3)	0.091
LGE: % of LV mass (%) if present	5.9 (2.9–8.8)	5.9 (3.3–5.9)	5.1 (2.9–9.9)	0.897
VO2 peak (mL/min/kg)	38.5 ± 11.3	38.3 ± 10.6	38.7 ± 12.1	0.895
NSVT, *n* (%) on 24 h ECG	4 (7.4)	2 (7.1)	2 (7.7)	1.00
ESC 5-year SCD risk score (%)	1.64 (1.34–2.32)	1.87 (1.47–3.67)	1.54 (1.24–2.20)	0.048

CV, cardiovascular; ECG, electrocardiogram; FH, family history; G+/G−, genetic positive/negative; HCM, hypertrophic cardiomyopathy; ICD, implantable cardioverter-defibrillator; LGE, late gadolinium enhancement; LV, left ventricular; LVEDD, left ventricular end-diastolic diameter; LVEF, left ventricular ejection fraction; NSVT, non-sustained ventricular tachycardia; SCD, sudden cardiac death; WT, wall thickness.

During a follow-up of 6.3 years (3.0–12.6) (*Table 2*), 19 participants (32.2%) experienced at least one cardiovascular event. Ventricular arrhythmic events occurred in 13 individuals (22.0%). Two ventricular fibrillation (VF) episodes were appropriately terminated by ICD therapy, without associated physical injury, one at rest and one during low-intensity exercise; one patient had previously presented NSVT during the follow-up period. No SCA/SCD occurred. Eleven patients had isolated NSVT (18.6%), only one symptomatic. Considering all NSVT cases, including the patient who subsequently experienced VF, NSVT was mainly detected during Holter monitoring (*n* = 9). Following NSVT detection, one patient underwent ICD implantation, and beta-blocker therapy was initiated or up-titrated in nine. Ventricular arrhythmic events occurred early during follow-up [3.9 months (1.4–7.5)]. AF occurred in four participants (6.8%), ischaemic stroke and hospitalization for heart failure occurred in one each (1.7%). At last follow-up, 52 participants (88.1%) were still engaged in sport activities.

**Table 2:** Follow-up results

	All participants (*n* = 59)	G+ group (*n* = 29)	G− group (*n* = 30)	*P*-value
Follow-up period (years)	6.3 (3.0–12.6)	6.1 (3.0–13.0)	6.4 (2.7–12.4)	0.638
Ventricular events, *n* (%)	13 (22.0)	6 (20.7)	7 (23.3)	0.807
NSVT, *n* (%)	11 (18.6)	4 (13.8)	7 (23.3)	
VF/ICD shock, *n* (%)	2 (3.4)	2 (6.9)	0 (0.0)	
SCA/SCD, *n* (%)	0 (0.0)			
All cardiovascular events, *n* (%)	19 (32.2)	9 (31.0)	10 (33.3)	0.850
Atrial fibrillation, *n* (%)	4 (6.8)	3 (10.3)	1 (3.3)	
Ischaemic stroke, *n* (%)	1 (1.7)	0 (0.0)	1 (3.3)	
Hospitalization for heart failure, *n* (%)	1 (1.7)	0 (0.0)	1 (3.3)	
Sport activity at last follow-up, *n* (%)	52 (88.1)	27 (93.1)	25 (83.3)	0.424

G+/G−, genetic positive/negative; ICD, implantable cardioverter-defibrillator; NSVT, non-sustained ventricular tachycardia; SCA/SCD, sudden cardiac arrest/death; VF, ventricular fibrillation.

### Comparisons between G+ and G− groups

Overall, the two groups were largely comparable. However, G+ participants were younger, more frequently had a first-degree relative with a history of HCM, and less commonly exhibited an apical phenotype. ESC HCM risk-score was slightly higher in the G+ group. Sports participation characteristics were similar between groups.

**Figure 1:**
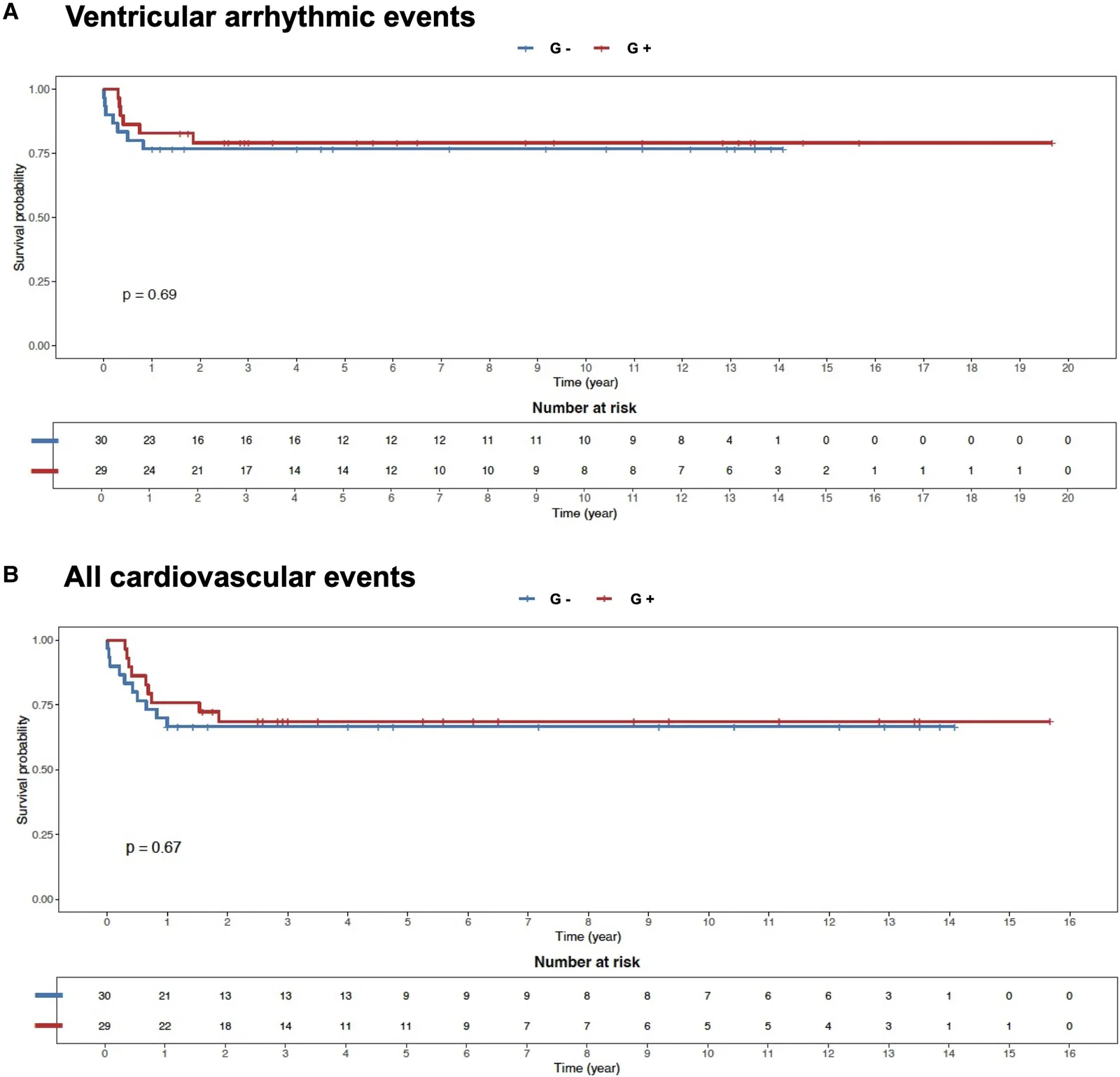
Clinical outcomes according to genetic status. (*A*) Ventricular arrhythmic events. (*B*) All cardiovascular events. G+: genetic positive; G−: genetic negative.

Ventricular arrhythmic event rates were similar between groups (20.7% in G+ vs. 23.3% in G−, *P* = 0.807), with comparable event-free survival (log-rank *P* = 0.685) (*Figure 1*). When NSVT was excluded, hard ventricular arrhythmic events were limited to the two appropriate ICD therapies for VF in G+ patients (6.9% in G+ patients vs. 0% in G− patients, *P* = 0.237). The overall occurrence of cardiovascular events was also similar between groups (31.0% in G+ vs. 33.3% in G−, *P* = 0.850), with no difference in event-free survival for the composite endpoint (log-rank *P* = 0.674).

## Discussion

In this exploratory cohort of individuals with low-risk HCM practicing regular sports activities, ventricular arrhythmic and cardiovascular event rates were similar between G+ and G− participants during follow-up. These findings align with studies suggesting that genotype status, although informative in defining disease causality, does not reliably discriminate individual arrhythmic risk in HCM.^5,6^

Whether sports participation amplifies arrhythmic risk in HCM remains a central question in sports cardiology. Contemporary studies, including the prospective LIVE-HCM study, consistently show that structured or vigorous exercise does not increase major cardiac events in appropriately selected HCM patients.^7–9^ Our findings are consistent with this evidence base. Although 22% of participants exhibited ventricular arrhythmias, nearly all were NSVT. Hard ventricular arrhythmic events were limited to two VF appropriately treated by an ICD therapy, and no SCD occurred during more than six years of follow-up despite continued sports participation in 88% of the cohort.

The inclusion of NSVT in the primary endpoint should be interpreted with caution. NSVT is not equivalent to hard arrhythmic events such as sustained ventricular arrhythmia, appropriate ICD therapy, and SCA/SCD. However, in sports-active HCM patients, NSVT remains clinically relevant as a marker of ventricular arrhythmic expression and may influence individualized counselling regarding sports participation. In the present cohort, NSVT detection also influenced clinical management, leading to ICD implantation in one patient and initiation or up-titration of beta-blocker therapy in nine patients. These findings suggest that arrhythmic events likely reflected the underlying HCM substrate rather than sport-induced electrical instability.

### Limitations

This study is limited by its retrospective design and the low number of hard ventricular arrhythmic events, most arrhythmic events consisting of NSVT. In addition, several baseline differences between G+ and G− participants, including age, apical phenotype, family history of HCM, and baseline ESC 5-year SCD risk score, may have influenced outcome comparisons and limit causal interpretation. The evolution of genetic panels over the study period may also have influenced genotype classification. These findings should therefore be considered exploratory and require confirmation in larger cohorts.

## Conclusion

In this exploratory cohort of sports-active HCM, ventricular arrhythmic and cardiovascular event rates were similar between G+ and G− participants. Hard ventricular arrhythmic events were rare, and no SCA/SCD occurred.

## Data Availability

The data underlying this article will be shared on reasonable request to the corresponding author.
